# Restoration of angiogenic capacity in senescent endothelial cells by a pharmacological reprogramming approach

**DOI:** 10.1371/journal.pone.0319381

**Published:** 2025-02-28

**Authors:** Katrin Kalies, Kai Knöpp, Susanne Koch, Claudia Pilowski, Leonie Wurmbrand, Daniel Sedding

**Affiliations:** Mid-German Heart Center, Department of Internal Medicine III, Division of Cardiology, Angiology and Intensive Medical Care, University Hospital Halle, Martin-Luther-University Halle-Wittenberg, Halle (Saale), Germany; Virginia Commonwealth University, UNITED STATES OF AMERICA

## Abstract

Senescent endothelial cells (EC) are key players in the pathophysiology of cardiovascular diseases and are characterized by a reduced angiogenic and regenerative potential. Therefore, targeting these cells has been suggested as an effective therapeutic strategy to reduce vascular disease burden and potentially improve health and lifespan of humans. Here, we aimed to establish a pharmacological, partial reprogramming strategy to improve replicative senescent endothelial cell function in the context of angiogenesis. We demonstrate that our treatment improves tube formation and sprouting capacity but also increases proliferation and migration capacity *in vitro*. Further, inflammation and DNA damage were reduced in the replicative senescent cells. These processes were initiated by a short and timely-restricted overexpression of the Yamanaka-factors induced by our pharmacological strategy. The advantage of these compounds is that they are FDA approved in their respective concentrations which could pave the way for use in a clinical setting.

## Introduction

Vascular function is highly impaired during aging, and vascular dysfunction is the underlying cause of cardiovascular diseases, the leading cause of death worldwide [1–3]. Clinically these alterations are among others observable by an increased systolic blood pressure [1,4] and increased size and stiffness of the large arteries [1]. Recent studies indicate that these alterations mainly result from a dysfunctional endothelium developing with advancing age [5]. The existence of senescent endothelial cells can draw a causal correlation between aging, endothelial dysfunction and cardiovascular diseases [4]. Senescent cells accumulate over the life span in the vasculature [6,7], in older healthy humans [1] and in diseased tissue in the pathogenesis of heart failure [8] or ischemic heart disease [9]. Senescent cells are stopped in the G0/G1 phase of the cell cycle [10,11] and negatively impact several downstream pathways such as inflammation, DNA damage, molecular regulators as well as cell cycle regulation [12]. Together, these processes lead to an impaired function of the aged endothelium by mainly minimizing the angiogenic and regenerative potential [13–16]. This makes reversing and rejuvenating senescent endothelial cells a highly interesting target to improve vascular regeneration in old individuals. Currently, different approaches to counteract cellular senescence are under intensive investigation, such as the use of senolytics to induce cell death or the process of cellular reprogramming mainly relying on viral transduction [12]. The concept of cellular reprogramming was originally used to illustrate the transformation of somatic cells into induced pluripotent stem cells using retroviral overexpression of OCT3/4, SOX2, KLF4, and c-MYC [17]. This reprogramming has exhibited therapeutic promise and has also indicated the potential to reverse aging-related traits, particularly evident in initial experiments conducted on senescent and centenarian cells [18,19]. However, prolonged induction of OSKM using viral methods in living organisms has led to teratoma formation and changes in DNA methylation patterns [20,21]. In the context of aging and cellular senescence, the term cellular reprogramming has been more commonly associated with the rejuvenation process of senescent cells rather than the generation of pluripotent stem cells [22,23].

Here, we aimed to develop a pharmacological strategy to improve senescent endothelial cell function, especially in the context of angiogenesis. Recently, a cocktail of small pharmacological compounds was presented, that contributed to liver regeneration and hepatic function *in vivo* by promoting cellular reprogramming [24]. This cocktail is composed of three compounds, namely tranilast, valproic acid (VPA) and lithium carbonate. All three substances are not only described for their supporting regenerative effect but also for beneficial effects on aging [24–29]. Indeed, here we are the first to demonstrate that the cocktail favors a reversion of the EC senescent phenotype *in vitro*. Importantly, all three substances are FDA-approved drugs already in use in clinical settings or at least clinical trials [30–36] simplifying a potential transition from bench to bedside.

## Materials and methods

### Cell culture and pharmacological reprogramming

All *in vitro* experiments were performed on human umbilical vein endothelial cells purchased from Lonza. Cells were cultivated in endothelial growth medium (PromoCell) at 37°C with 5% CO_2_ in a humidified incubator, grown to confluency, and passaged in a 1:3 ratio. Every batch used was previously tested for their (early) senescent cell state as recently described [37]. Based on these criteria the used passages for each batch in the experiments were determined, which was generally but batch-dependent below passage 5 for non-senescent cells and above passage 14 for replicative senescent cells. For the pharmacological reprogramming cells were treated with 0.3 mM Li_2_CO_3_ (Sigma, solved in water), 0.5 mM valproic acid (Sigma, solved in water), and 30 µM Tranilast (Selleckchem, solved in DMSO) for 72 hours. After the treatment cells were cultivated for additional 7 days to measure cellular function and assess cellular senescence on day 10 after the treatment. The controls, here replicative senescent untreated and non-senescent cells, were treated with the solvents in the respective concentration.

### siRNA transfection experiments

siRNA-Transfection was performed using Lipofectamine RNAiMax (Thermo Fisher Scientific) according to the manufacturer’s protocol. The respective siRNA was mixed with Lipofectaime RNA iMax and shortly incubated in Opti-MEM media (Gibco, Thermo Fisher Scientific). Cells were seeded in antibiotic-free media and incubated with 20nM of the siRNA complexes. Efficiency of siRNA-transfection was assessed by qRT-PCR. Information on used siRNA can be found in S1 Table.

### RNA isolation, reverse transcription and qRT-PCR

Total RNA was isolated using the Plus RNeasy Mini Kit (Qiagen) according to the manufacturer’s protocol. Briefly, cells were washed and lysed and RNA was isolated on a column based-isolation including removement of genomic DNA. RNA was transcribed to cDNA using the High Capacity cDNA Reverse Transcription Kit (Thermo Fisher) and qRT-PCR analysis was performed with the Blue S’Green qPCR Kit (Biozym). Both kits were used according to the manufacturer’s instructions with an input of 100 ng total RNA. A list of used primers is shown in S2 Table.

### DNA isolation and determination of telomere length

Genomic DNA was isolated following the manufacturer’s instruction with the GeneJET Genomic DNA Purification Kit (Thermo Fisher Scientific) which works on a column-based isolation technique including treatment with Proteinase K and RNase A. Telomere length was determined via qRT-PCR analysis based on the Relative Human Telomere Length Quantification assay (ScienCell Research Laboratories).

### Proliferation and migration

Proliferation was assessed in a life-cell imaging approach. Images of sub-confluent seeded cells were taken over a period of 24 hours and cells were counted automatically in every brightfield image. Further, cell growth was evaluated using a Bromodeoxyuridine (BrdU) assay (Cell proliferation ELISA - colorimetric, Roche), following the guidelines provided by the manufacturer. Absorbance was measured at 450nm in reference to 690 nm at a plate reader.

Migration capacity was determined by a scratch-wound assay. Cells were seeded to confluency and a scratch wound was introduced with a pipette tip. Images were taken every 30 minutes over 24 hours. Cell-Area was calculated to determine migration capacity.

All experiments were performed with the life cell imaging system Cytation 1 (Biotek) and image analysis was performed with the software Gen 5 (Biotek).

### In vitro angiogenesis assays

Two different angiogenesis assays were performed *in vitro,* a sprouting assay and a tube formation assay. For the sprouting assay cells were seeded with a density of 4 * 10^cells/ml in 80%growth-medium and 20% methylcellulose into hanging droplets for 24 hours to allow sphere formation. On day 2 a bottom matrix was pre-prepared (50% of 2 mg/ml Collagen, 10% M199 Medium and 40% Methylcellulose-Stock solution) and polymerization was allowed for 1 hour at 37°C. For the top matrix, spheroids were collected, centrifuged and resuspended in FCS and further mixed with 40% Methylcellulose and 50% Collagen. Polymerization was allowed for 2 hours, then the matrix was overlaid with the medium. The formation of sprouts was controlled by microscopy.

The tube formation assay was performed using the “In vitro Angiogenesis Assay Kit” from Cultrex according to the manufacturer’s protocol. Briefly, plates were coated with a basement membrane followed by a polymerization step. Up to 10.000 cells were seeded into one well of the plate and the plate was further incubated in the life cell imaging system Cytation 1 to observe the formation of tubular networks. Tube formation capacity was assessed 8 hours after seeding the cells.

### Immunofluorescence staining

Immunofluorescence stainings were performed on *in vitro* cultivated cells. Slides were fixed for 30 min, blocked and incubated with the primary antibody overnight. On day 2, slides were washed and incubated with the secondary antibody for 2 hours. After additional washing steps, the slides were embedded in DAPI and closed with a cover slip and sealed. For microscopy a Zeiss-Axio Observer was used. Quantification was performed by analyzing multiple fields of view per each technical replicate per independent biological replicate. Image analysis was performed with Fiji-ImageJ [38]. For CD31-analysis, CD31-positive stained cells were counted in relation to the total number of cells indicated by DAPI. Analysis of nuclei size was performed by quantifying the area of positive DAPI-stained signals per image. A list of used antibodies is presented in S3 Table.

### Fluorescence-based senescence-associated β-galactosidase staining

Fluorescence-based senescence-associated β-galactosidase staining was performed as previously described by Debacq-Chainiaux et al. [39]. Attached cells were treated with 100 nM bafilomycin A1 for 1h at 37°C in 5% CO_2_ in fresh culture medium to ensure lysosomal alkalinization. Then, C_12_FDG was added to the cells in a final concentration of 33 µM to ensure staining of β-galactosidase activity. Staining was visualized by fluorescence microscopy (Zeiss). At least 3 fields of views per technical replicate (here 4) in each biological replicate were analyzed by counting b-gal positive stained cells (green) in relation to the total number of cells. Image analysis was performed using Fiji-ImageJ. Images were chosen as representative images for the C12FDG staining, not for cell morphology, as it is influenced by the bafilomycin A1 treatment to induce lysosomal alkalinization.

### Statistical analysis

Datasets were analyzed using GraphPad Prism 8. All data are represented as mean ± standard deviation, n = 3 indicates the number of individual experiments performed (biological replicates). All datasets were normalized to a control group (non-senescent cells) for fold change analysis and tested for variance. Unpaired Student’s t-test and one-way ANOVA were performed. ANOVA was employed to compare multiple groups to assess statistical differences whereas unpaired t-tests were used for pairwise comparisons. The probability of error is described in each individual figure.

## Results

### Combinational treatment of senescent endothelial cells with valproic acid, lithium carbonate and tranilast does not lead to morphological alterations but improves proliferation

**Fig 1:**
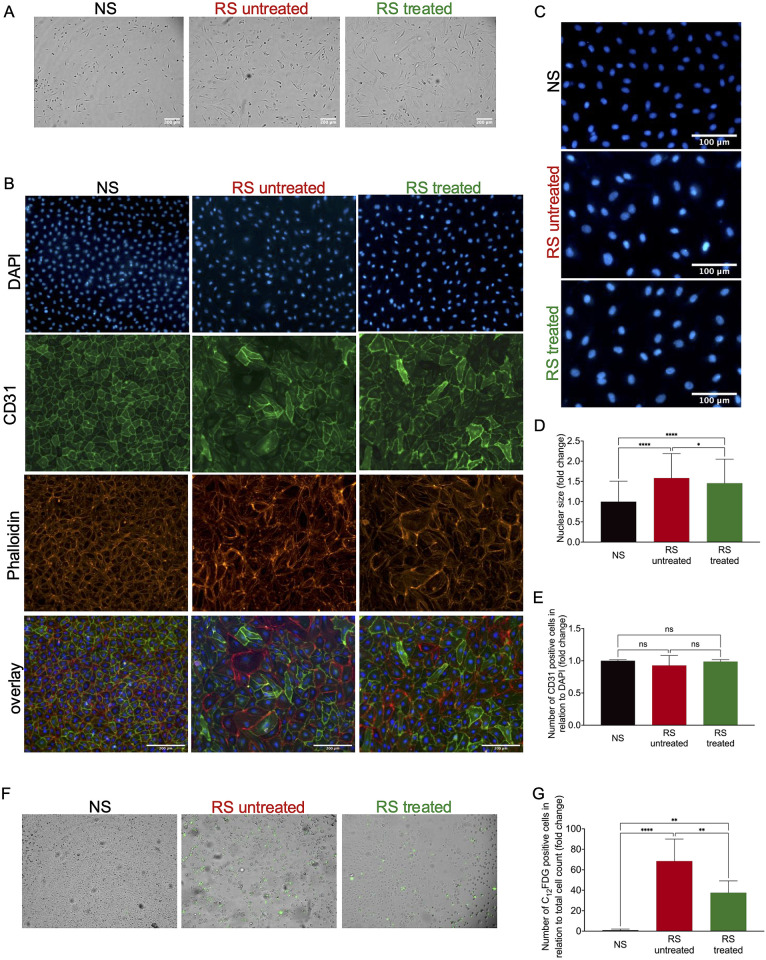
Pharmacological treatment does not influence endothelial cell identity or cell size but decreases the nuclear size. (A) Brightfield images of non-senescent (NS), replicative senescent untreated (RS untr) and replicative senescent treated (RS treated) cells; (B) Immunofluorescent staining of non-senescent (NS), replicative senescent untreated (RS untreated) and replicative senescent treated (RS treated) cells for DAPI (blue; cell nuclei), CD31 (green; endothelial cell marker) and Phalloidin (red; F-Actin for cellular size); (C) Zoom image of the DAPI staining of non-senescent (NS), replicative senescent untreated (RS untreated), and replicative senescent treated (RS treated) cells to quantify nuclear size; (D) Quantification of nuclear size by measurement of the area of DAPI staining with Fiji-ImageJ. (NS ± 790µm^2^; RS untreated ± 1260µm^2^, and RS treated ± 1150µm^2^). n = 3 *p < 0.05, ****p < 0.0001; (E) Quantification of the number of CD31-positive stained cells to total cell number by DAPI-staining to assess EC identity. n = 3; (F) Fluorescence-based senescence associated ß-galactosidase staining (green) of non-senescent (NS), replicative senescent untreated (RS untreated) and replicative senescent treated (RS treated) cells. Images are chosen as representative images for the C12FDG staining, not for cell morphology as it is influenced by the experimental setup. (G) Quantification of C12FDG positive cells in relation to total cell number (right). n = 3, **p < 0.01, ****p < 0.0001.

First, the effect of the pharmacological cocktail on cellular morphology was studied by brightfield imaging and immunofluorescence stainings. Replicative senescent endothelial cells presented with the expected specific morphology with increased cell and nuclear size. Treatment with the pharmacological cocktail did not lead to any morphological changes in regard to cell size (Fig 1A and 1B). However, the size of cell nuclei was significantly smaller in the treated cells, but not comparable with non-senescent EC (Fig 1C and 1D). Furthermore, cells of all three conditions presented with a positive CD31 co-staining (Fig 1B and 1E), as well as vWF, CD146, and VE-cadherin (S1 Fig), confirming the endothelial cell identity. The senescence associated ß-galactosidase staining showed fewer positive cells for the treatment group than for the untreated control (Fig 1F and 1G).

Senescent cells always exist in the direct neighborhood of non-senescent cells in the vasculature. So, an important aspect of a potential intervention is that the treatment does not lead to negative aspects on non-senescent cells. We, therefore, reperformed measurement of proliferation on treated non-senescent cells according to the same protocol. Also, the treatment did not affect non-senescent cells’ endothelial cell identity (S2A Fig).

**Fig 2:**
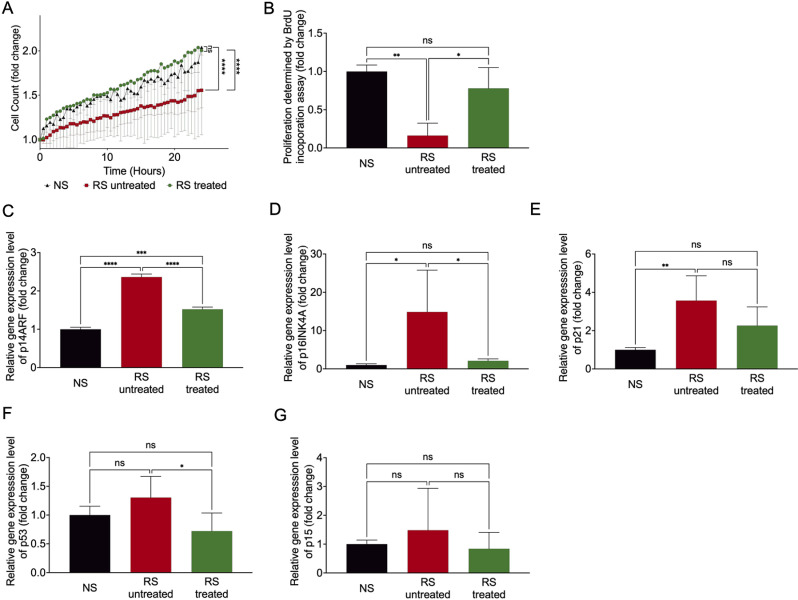
A cocktail of VPA, Li_2_CO_3_, and tranilast can improve the proliferation, influences cell cycle regulators and production of mitochondrial superoxide of replicative senescent EC. (A) Determination of proliferation by total cell count in live-cell imaging over 24 hours. Cell count is expressed as fold change to time point 0h. n = 3 ****p < 0.0001; (B) Quantification of proliferation determined by BrdU incorporation assays. n = 3 **p < 0.01, *p < 0.05; (C–G) Quantification of p14ARF, p16INK4A, p21, p53 and p15 mRNA-expression by qRT-PCR. n = 3 *p < 0.05, **p < 0.01, ***p < 0.001, ****p < 0.0001.

Next, the effect of the pharmacological cocktail on proliferation and metabolic activity was assessed. As expected, replicative senescent endothelial cells had a highly impaired proliferation rate in comparison to non-senescent cells as determined by live cell counting over 24 hours (Fig 2A). In contrast, the pharmacological cocktail provoked a significant improvement in the treated cells. Results could be confirmed by a BrdU incorporation assay (Fig 2B). So far, we could show that the cocktail did not affect cell proliferation either by total cell count in live-cell imaging nor in the BrdU (S2B and S2C Fig). No significant differences between the non-senescent cells receiving the pharmacological treatment and those not receiving the treatment were measurable. In line with the improved proliferative effect in the senescent cells, we measured a significant decrease of p14ARF, a prominent cell cycle regulator in senescence on mRNA expression level in the senescent cells (Fig 2C). This goes in line with significantly reduced p16INK4A and p53 mRNA-expression levels in the replicative senescent treated cells whereas level of p21 was not affected (Fig 2D–2F). The cell cycle gene p15 was not regulated in either condition (Fig 2G).

### The pharmacological cocktail reduces inflammation and improves angiogenic capacity in vitro

**Fig 3:**
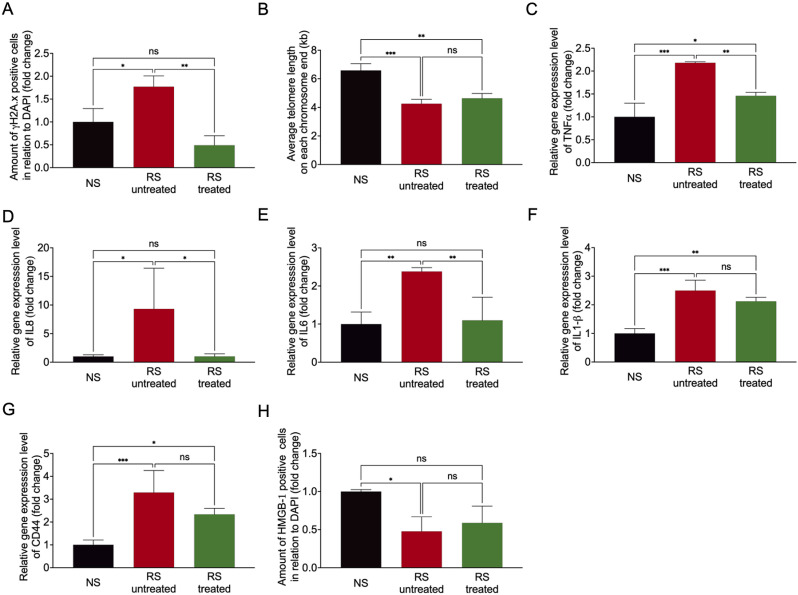
Common characteristics of replicative senescent cells can be reversed by pharmacological treatment. (A) Quantification of DNA double-strand breaks by γH2Ax staining relative to DAPI. n = 3 **p < 0.01 *p < 0.05; (B) Measurement of average telomere length by gDNA isolation and qRT-PCR. n = 3 **p < 0.01, ***p < 0.001; (C–G) Quantification of the mRNA expression level of TNF-α, IL8, IL6, IL1-ß and CD44 by qRT-PCR. n = 3 *p < 0.05, **p < 0.01, ***p < 0.001; (H) Quantification of HMGB-1 release by immunofluorescence staining in relation to DAPI. n = 3 *p < 0.05.

Another important feature of senescent cells is the initiation of the DNA damage response induced by DNA double-strand breaks and a reduction of the telomeres at the end of chromosomal DNA. The senescent cells treated with the cocktail had significantly less DNA double-strand breaks, as quantified by γH2A.x staining, than the untreated cells (Figs 3A and S3A). Further, telomere length was not affected by the pharmacological treatment (Fig 3B).

The release of growth factors and cytokines as part of an inflammatory process plays an important role in the existence of senescent cells, known as the senescence-associated secretory phenotype. Among these cytokines are interleukin-1ß (IL-1ß), tumor necrosis factor-α (TNF-α), Interleukin-6 (IL6), Interleukin 8 (IL8), and CD44 all factors especially known for endothelial cell senescence, and the molecular regulator High-Mobility-Group-Protein B1 (HMGB-1). Here we show a rescue reduction of gene expression in senescent cells almost to non-senescent cell levels for TNF-α, IL6, and IL8 by the pharmacological cocktail. Expression of all three genes was significantly decreased by the treatment (Fig 3C–3E). Expression levels of CD44 and IL1- ß were not affected (Fig 3F and 3G). In addition, HMGB-1 levels were not yet affected by the treatment (Figs 3H and S3B).

**Fig 4:**
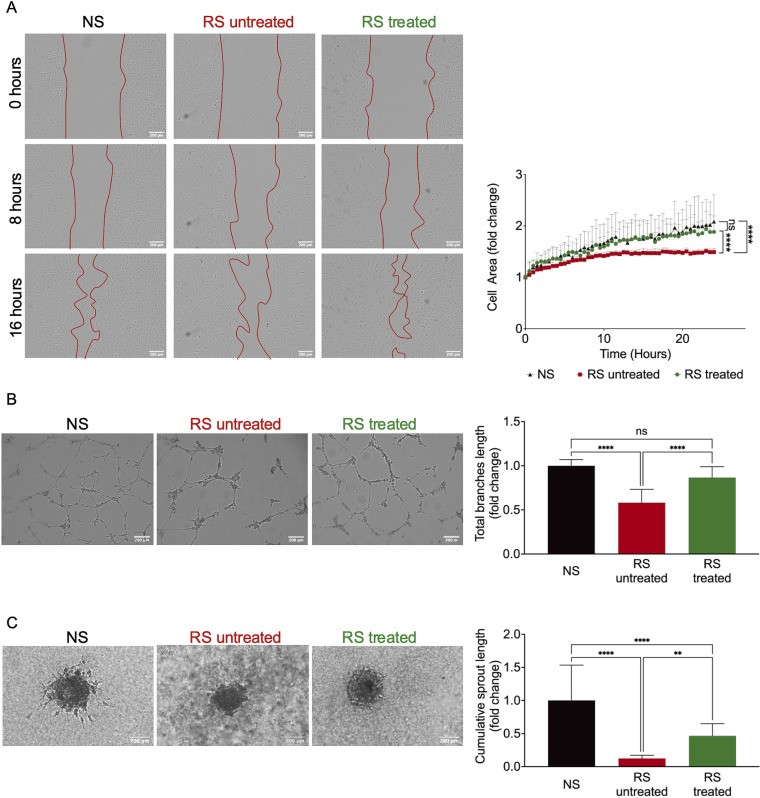
The combination therapy with VPA, Li_2_CO_3_, and tranilast can restore the angiogenic capacity of replicative senescent EC. (A) Determination of migration capacity by a scratch-wound assay. Exemplary images 0, 8 and 16 hours after scratch-wound. n = 3 ****p < 0.0001; (B) Determination of angiogenic capacity by a tube formation assay and quantification of total branches length. n = 3 ****p < 0.0001; (C) Endothelial cell sprouting assay and cumulative sprout length as a measure of angiogenic capacity. n = 3 **p < 0.01, ****p < 0.0001.

In addition to standard pathways that are dysregulated in senescent endothelial cells, the effect of the pharmacological cocktail on essential endothelial cell function was investigated. First, endothelial cell migration capacity was studied by a scratch-wound assay. Scratch closure occurred faster in the senescent treated cells than in the untreated cells back to non-senescent control levels (Fig 4A). Further, the angiogenic capacity of the cells was tested *in vitro* by a tube formation assay, a 2D- assay were the ability of the cells to form capillary-like structures is assessed, and an endothelial sprouting assay. In contrast, she sprouting assay is performed in a 3D-environement, where cells degrade the surrounding matrix and invade it [40–42]. Both assays showed an improved angiogenic capacity in the senescent cells receiving the pharmacological cocktail. Total branch length (Fig 4B) and cumulative sprout length (Fig 4C) were significantly longer in treated senescent cells, almost back to non-senescent EC levels. Further, quantification of angiogenic markers as ANG-1 and VEGF showed an up-regulation by the treatment (S4 Fig). In the non-senescent cells, treatment with the pharmacological cocktail did not affect migration capacity or tube formation capability (S2D and S2E Fig).

### Initiation of regeneration by overexpression of the Yamanaka-factors might lead to functional improvement

**Fig 5:**
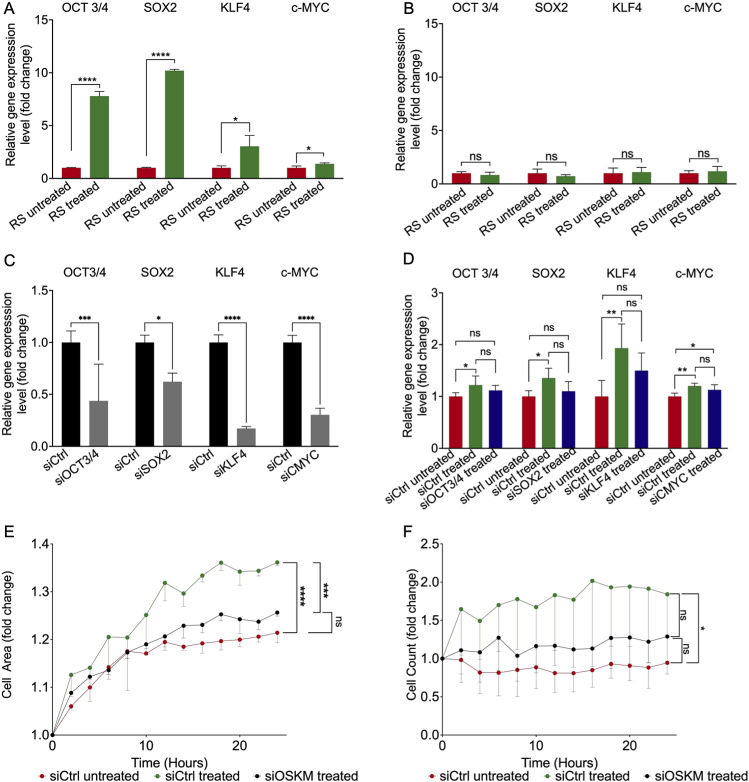
The treatment with VPA, Li_2_CO_3_, and tranilast leads to a short-time overexpression of the Yamanaka-factors OCT3/4, SOX2, KLF4, and c-MYC. (A) Quantification of the relative gene expression level of OCT3/4, SOX2, KLF4, and c-MYC directly after the 72 hours of treatment. n = 3 *p < 0.05, ****p < 0.0001; (B) Quantification of the relative gene expression levels of OCT3/4, SOX2, KLF4, and c-MYC was performed 7 days following the 72-hour treatment period. n = 3; (C) Quantification of the efficiency of siRNA mediated gene knockdown on mRNA expression level OCT3/4, SOX2, KLF4, and c-MYC by qRT-PCR. n = 3 *p < 0.05, ***p < 0.001, ****p < 0.0001; (D) Quantification of the relative gene expression level of OCT3/4, SOX2, KLF4, and c-MYC after respective siRNA transfection and the 72-hours treatment with VPA, Li_2_CO_3_, and tranilast. n = 4 *p < 0.05, **p < 0.01; (E) Determination of migration capacity by a scratch-wound assay after respective siRNA transfection and the treatment with VPA, Li_2_CO_3_, and tranilast. n = 3 ***p < 0.001, ****p < 0.0001; (F) Determination of proliferation by total cell count in live-cell imaging over 24 hours after respective siRNA transfection and the treatment with VPA, Li_2_CO_3_, and tranilast. Cell count is expressed as fold change to time point 0h. n = 3 *p < 0.05.

To gain more mechanistical insight into working of the pharmacological cocktail, we decided to further study the initiation of regeneration in the senescent-treated cells. All three pharmacological compounds have already been shown to initiate the regenerative effect and support traditional cellular reprogramming strategies by viral vectors that are based on the overexpression of the Yamanaka-factors, OCT3/4, SOX-2, KLF-4, and c-MYC (combined: OSKM). These methods harbor the risk of genomic instability and teratoma formation. Thus, we studied the expression of the Yamanaka-factors at different time points after the treatment to first see if these factors are the leading cause of functional recovery. All four factors were significantly enhanced 72 hours after the treatment (Fig 5A). In contrast, the expression of the factors dropped to a normal physiological level as soon as the substances were removed from the cells (Fig 5B). Interestingly, we could verify that in the non-senescent cells, upregulation of OSKM cannot be observed on day 3 as in the replicative senescent cells and also to no other time point (S2F and S2G Fig). By this, an important criterion for an intervention to target senescent cells was cleared. In order to further verify the interplay between the treatment, OSKM induction, and functional improvements, siRNA experiments to knockdown OSKM were performed in replicative senescent cells. Given the assumption that siRNA transfection results in decreased activity of OSKM, while administering the cocktail leads to their increase, we anticipated that the combined effect of siRNA downregulation and cocktail treatment would counteract each other, aiming to maintain gene expression comparable to the control group. First, the efficiency of siRNAs transfection was quantified, showing a significant downregulation of OSKM (Fig 5C). Next, siRNA-mediated knockdown of the four genes parallel to the treatment with the pharmacological cocktail was performed to study the interplay of the knockdown and simultaneous stimulation by the cocktail. Indeed, in the siRNA treated samples, overexpression of OCT3/4, SOX-2, and KLF-4 was prevented and did not differ from the untreated siCtrl group (Fig 5D) showing the expected counteracting effect. For c-MYC relative gene expression was still slightly enhanced in siMYC treated cells, but the upregulation was lower than for siCtrl treated cells in comparison to the siCtrl untreated cells. To further assess if the effects of the treatment on cellular function are influenced by the absence of these factors, migration and proliferation after siRNA transfection and treatment of the senescent cells was studied. Scratch closure and proliferation of treated cells that were transfected with siRNA for all four genes (siOSKM) was not improved and remained at the same level as the untreated controls (Fig 5E and 5F) Further, also the single knockdown of one factor of OSKM was sufficient to prevent the pro-migratory and pro-proliferative effect of the pharmacological cocktail (S5 Fig).

## Discussion

Endothelial dysfunction is a key driver of cardiovascular diseases provoked by external noxa and inflammation, and the occurrence of senescent cells in the vasculature [4–7]. These senescent endothelial cells set limits to the regenerative and angiogenic capacity of the vasculature [13–16]. To overcome this hurdle and potentially improve the outcome of CVDs, we aimed to target these senescent endothelial cells and improve cellular function. Here we show, that a combinational application of the substances VPA, Li_2_CO_3_, and Tranilast improves cell proliferation, partially reflected at cell morphology level, restores mitochondrial superoxide, and reduces inflammation. Further, the treatment did not affect telomere length and DNA damage and significantly improved the angiogenic capacity of replicative senescent cells. All of these improvements were likely initiated by a short and timely-restricted overexpression of the Yamanaka-factors OCT3/4, SOX2, KLF4, and c-MYC.

So far, little is known about promising intervention options for senescent cells. In general, among these options is to enhance the immune response [12,43,44] or the replicative potential of the cells, for example by prolonging telomere length [12,45]. In addition to that, the main focus is on the senolytics, substances that can induce the cell death of senescent cells [12]. This approach has already been shown to delay aging in a mouse model of accelerated aging by clearing p16INK4A-positive cells [46]. In contrast, recent studies show that the elimination of senescent cells defined by high p16INK4A expression contributes to problems regarding tissue structure and function [47]. Furthermore, combinational therapy of dasatinib and quercetin or the application of ABT263 showed reversal of the aged phenotype [48,49].

As the latest option, cellular reversion or reprogramming is under investigation to interact with senescent cells [12]. Until now the term cellular reprogramming was mainly known for reprogramming somatic cells into induced pluripotent stem cells by retroviral overexpression of OCT3/4, SOX2, KLF4, and c-MYC [17]. Cellular reprogramming has already been shown to be useful as a therapeutic tool in different cell types [50] and also had first experimental setups working with senescent and centenarian cells [18], hinting towards a potential reversion of the aged phenotype [19,22]. Further, it was shown that the generation of induced vascular progenitor cells from endothelial cells by a partial OSKM overexpression is possible and that they can be differentiated into vascular smooth muscle cells and endothelial cells [51]. All these approaches worked with a viral overexpression of the factors harboring the risk of genomic instability and teratoma formation [19]. Indeed, it was shown that a virally induced long-term induction of OSKM *in vivo* led to teratoma formation and changed DNA methylation patterns [20,21]. Here, we demonstrate that the induced overexpression of OSKM is also achievable by small chemical compounds, that are easier to control in a time- and concentration-dependent manner as well as in application [52,53]. It was already shown that transient reprogramming by cyclic induction of OSKM in an OSKM-inducible mouse strain already showed promising reversal of the aged phenotype and increased lifespan [23]. Further, it has been demonstrated that fibroblasts and endothelial cells obtained from patients can ameliorate age-related traits through the temporary and non-permanent activation of nuclear reprogramming factors OSKM [54]. The transient overexpression of OSKM shown here for such a short time seems not to be long enough to generate iPSC. This is confirmed by the exclusiveness of the combination of the endothelial cell markers[55,56], which are not expressed in stem cells and therewith show that cell keep their endothelial identity and are not induced to pluripotency. However, the partial regulation of OSKM might achieve partial reprogramming and therewith already a functional improvement.

The pharmacological cocktail used here consists of valproic acid, lithium carbonate, and tranilast. All compounds are FDA-approved in their respective concentration, enabling a further transition into clinical settings. Further, all three compounds are not only known to support cellular reprogramming but are also shown to have a potential role in cellular aging processes by interacting with aging-related pathways [24–29]. Valproic acid as a histone deacetylase inhibitor supports the development of neural stem and progenitor cells [29,57] and reduces common mechanisms of aging shown in *C. elegans* [26]. A potential target of VPA is the extracellular signal-related kinase pathway (ERK) which in turn is discussed to be associated with aging and promotion of cellular senescence and inhibits apoptosis in endothelial cells [58,59]. Lithium carbonate, a glycogensynthase-kinase 3 (GSK-3) inhibitor, enhances iPSC generation [60–62] and blocks the formation of a GSK-3/p53 complex inhibiting senescence accumulation [27,28]. The formation of this complex here might not only be blocked by the inhibition on GSK-3, but also by the downregulation of p53. Tranilast mainly acts as TGF-ß inhibitor [63,64], as activation of TGF-β might block reprogramming [65]. Further, TGF-ß is known to play a role in aging for example in telomere function and cell cycle regulation therewith contributing to cellular senescence [66]. In turn, tranilast can support the generation of progenitor cells [29] and has anti-inflammatory [67,68], anti-oxidative [69], anti-allergic [70], and anti-fibrotic effects [71]. It also seems to have a non-beneficial effect on cell migration and angiogenesis [72,73]. So far, by literature research, we could not gain insights into the detailed respective function of the components in the context of endothelial cell senescence and their respective targeting of OSKM. We assume that every substance individually regulates one or several of the Yamanaka-factors OSKM leading to different expression levels of the genes after the treatment and thereby contributing to the improved cellular functions. Besides the role of OSKM in the regenerative process to maintain stem cell pluripotency and self-renewal, the genes also act as transcription factors and are crucial for both physiological cell functions and pathological processes. The exact mechanisms of how they regulate cellular functions is still under investigation. All four factors can, depending on the context either promote or inhibit proliferation in differentiated cells. KLF4 for example can inhibit proliferation by the regulation of cell cycle associated genes in the G1/S phase [74–78]. Further, SOX2, KLF4 and c-MYC are particularly important in regulating migration and invasion, often through pathways related to EMT, with implications in both development and cancer metastasis [78–80]. In addition, c-Myc and KLF4 are directly involved in angiogenesis, primarily by regulating the expression of VEGF and other angiogenic factors [76,81,82]. Especially KLF-4 seems to switch its role from pro-survival to pro-cell death in dependency of the cellular conditions. These observations indicate that OSKM can support the here observed functional improvements. That the substances used here indeed interplay with OSKM was verified by the siRNA experiments. The use of siRNA knockdown to counteract treatment-induced OSKM induction provided critical mechanistic insight into the role of this pathway in mediating the observed effects. By specifically and simultaneously suppressing OSKM the functional improvement, here as a pro-migratory and pro-proliferative effect, was prevented. This provides further evidence that OSKM activation is a central mechanism underlying the functional recovery induced by the treatment. Moreover, the observation that the knockdown of a single OSKM component is sufficient to counteract the positive functional aspect of the treatment shows that each of the factors is necessary for the here achieved regeneration. It can be assumed that there must be a complex interplay of OSKM and also in the regulation and mode of action of the pharmacological cocktail. However, we need to acknowledge the potential limitations of this study design as knockdown by siRNA was an incomplete silencing, and no off-target effects were studied which might influence the interpretation of the results. But, the usage of an siRNA-pool and consistent experimental outcomes strengthens the conclusion that OSKM regulation is indeed involved in this regeneration process.

The before mentioned aspects of the chemical compounds partially counterargument the experimental findings here. We could show that the angiogenic and regenerative capacity was restored *in vitro* by the pharmacological treatment. We suspect that the low dose of tranilast in combination with the other two factors enabled these improvements. We for example worked with an *in vitro* dosage of only 30 µM compared to other studies with over 70 µM showing inhibition of proliferation and tube formation [72].

Taken together our method of pharmacological OSKM induction not only reduces potential risk factors by avoiding viral vectors, but also by promoting only a partial, transient reprogramming strategy.

Due to the lack of data regarding partial reprogramming strategies in senescent EC, data presented here were usually compared to iPSC generation from (senescent) cells.

We could show that functional improvements regarding angiogenic capacity, proliferation, and migration capacity can be achieved by the treatment. The influence on the p53/p21 pathway by reduced p14ARF and p53 expression and the increased proliferation rate might be coherent. As a cyclin-dependent kinase, p14ARF is incorporated into the p53/p21 pathway. The regulator p53 is important for the G1/S checkpoint as well as the G2/M checkpoint. It is activated upon DNA damage and then prevents either DNA replication or mitosis. In the G1 phase, the activated p53 leads to the expression of p21, which further inhibits CDK2, causing cell cycle arrest. Expression of p14ARF leads to activation of p53 and p21 and thereby promotes cell cycle arrest and apoptosis [83–85]. Activation upon cellular or oncogenic stress, p14ARF inhibits MDM2, a negative regulator of p53, leading to the stabilization of p53 [83–86]. In contrast, p16INK4A can inhibit CDK4/6 and further prevent the phosphorylation of Rb. The unphosphorylated RB binds to E2F transcription factors, which would be necessary for the progression of to the S phase [83,84,86]. The two pathways, p14ARF/p53/p21/CDK2 and p16INK4A/CDK4/6 thereby collectively enforce checkpoints to prevent damaged or stressed cells from proceeding in the cell cycle. All these cell cycle markers, but especially p16INK4A and p14ARF, are commonly described to be responsible for the lack of proliferation of senescent cells [10,11,87]. We suspect the decreased expression of the cell cycle regulators to be responsible for the improved proliferation. Analysis of cell cycle phases might enforce these observations and further deepen the mechanistic insights. Similar findings for p16INK4A downregulation through the treatment were noted during the creation of induced pluripotent stem cells (iPSC) through viral cellular reprogramming of senescent cells, the generation of iPSC from fibroblasts, and employing a partial reprogramming approach involving the overexpression of OSKM via doxycycline induction *in vitro* [18,23,88]. Recent research indicates a notable direct relationship between senescence and OSKM-induced cellular reprogramming [89]. Cells that do not express the p16INK4A/ARF locus show reduced capability for reprogramming. Moreover, elevated levels of cellular senescence in naturally aging mice or in progeric mice promote OSKM-driven reprogramming [90]. Further, DNA damage can be restored with the treatment in the replicative senescent EC which is contrary to iPSC generation to reverse the aging phenotype where an accumulation of DNA damage and mutations in mitochondrial DNA was not restored by reprogramming [19,23]. The telomere length was stabilized by the pharmacological cocktail, a phenomenon known from iPSC generation where prolongation of telomeres is observable [18,91]. However, the lack of telomere elongation in connection with the improved proliferation must be viewed critically in view of the long-term effect of the treatment, as this could lead to increased apoptosis, a process similar to the application of senolytics. Senolytics are intended to promote tissue regeneration through the targeted apoptosis of senescent cells. Here, the direct entry of cells into apoptosis can be excluded by live-cell imaging in combination with BrdU, but later apoptosis would be possible. We assume that even if apoptosis occurs later, this has no negative consequences for the therapeutic effects. One explanation is that the initial enhancement of cellular regeneration and angiogenic capacity could support tissue repair and the recovery of surrounding cells, while subsequent apoptosis may further facilitate these processes, akin to the effects observed with senolytic treatments. Efficient reversal of SASP components as IL6, Il8 or TNF-α was observable as known from other reprogramming approaches such as for IL-6 [23]. Recent studies suggest that the crosstalk between senescence and reprogramming occurs through the cytokine-enriched microenvironment, primarily driven by IL-6. Consequently, this environment also promotes reprogramming in adjacent cells [90]. However, further investigations regarding the influence on inflammatory processes are needed as endothelial cells are only one of several key players in the regulation and participation of inflammatory processes [92,93]. HMGB-1 release is, among others factors, mediated by p53 [94], a factor which is also downregulated by the treatment. Though, levels of HMGB-1 were not affected by the pharmacological cocktail; an explanation might be that the time between the treatment and the assessment of HMGB-1 levels was not sufficient to restore intracellular HMGB-1 levels.

From a clinical perspective, also the ability to prevent endothelial cell senescence could have profound implications for vascular health and disease prevention, especially in at-risk patient populations. In our study, the treatment did not show significant effects on non-senescent endothelial cells, indicating that it may not influence the baseline state of healthy cells. This observation provides a first insight into the treatment’s potential to slow or prevent senescence; however, additional studies are required to evaluate its effects under senescence-inducing conditions. Interestingly, the lack of effect in non-senescent cells may also suggest a degree of specificity for senescent cells, which could limit off-target effects in therapeutic applications. Despite its promising effects on reversing senescence, the treatment activates regenerative pathways that include proliferation-associated genes, some of which may have oncogenic potential if chronically activated. This raises concerns about the safety of long-term treatment, particularly in non-senescent cells where such activation might lead to unregulated proliferation and tumor formation. Therefore, it will be essential to carefully balance the therapeutic benefits of the treatment with the potential risks of adverse effects.

In this exploratory pilot study, a small sample size (n = 3) was employed to investigate preliminary biological effects and establish foundational data. While statistical tests were utilized to identify potential trends, we acknowledge that the limited sample size may affect the statistical power and the generalizability of the findings. Small sample sizes are common in initial basic science laboratory research and serve to inform the design of subsequent studies to assess feasibility, optimize experimental conditions, and generate hypotheses. Future research with larger cohorts and *in vivo* experiments are necessary to validate these findings and provide more definitive conclusions, also in regard of detailed information on how OSKM are regulated and why.

In this study, we could show that the pharmacological cocktail worked well on replicative senescent EC as the main driver of endothelial dysfunction. However, it is of great interest to elucidate the effect of the cocktail on other cell types and potentially also on other organs as the cocktail is not cell type- or organ-specific. Here it is also important to look deeper into the regulation by the cocktail and why it for example does not regulate OSKM or cellular function in non-senescent endothelial cells. Another limitation might present in the effect size of the treatment. Interestingly, whereas functional improvements, such as proliferation and migration as well as expression of specific senesce-associated genes, could be substantially reduced almost entirely to a non-senescent level, the morphological changes and HMGB-1 did not show such an effect size. These changes, such as cell enlargement and cytoskeletal organization, are complex and may involve additional layers of regulation. However, taken together with the strongly improved angiogenic potential upon treatment, we assume a promising therapeutic potential by the treatment. Our findings indicate that the treatment successfully addresses critical functional deficits linked to cellular senescence, highlighting the need for further exploration in *in vivo* models. This transfer to an in vivo setting presents new limitations due to the pharmacokinetics and the associated distribution of the substances in the body and their cellular uptake. So far, the combination of the three compounds was also shown to promote cellular reprogramming and improve liver regeneration and hepatic function *in vivo* [24]. Their applied concentrations might offer a first orientation for an *in vivo* approach. In further experiments, the experimental design needs to be tested in a larger cohort including *in vivo* experiments to study regeneration and analysis to study the effects on a single cell level.

As a conclusion, this work emphasizes that targeting senescent endothelial cells as a driver of endothelial dysfunction is a promising therapeutic strategy. We could show that pharmacological treatment with VPA, Li_2_CO_3_, and tranilast restores functional properties of senescent endothelial cells such as proliferation, migration, and the angiogenic capacity *in vitro*. This mechanism might be initiated by the short and timely-restricted overexpression of OSKM. All in all, this process might open new translational perspectives in supporting vascular regeneration after acute or chronic cellular damage.

## Supporting information

S1 Fig Immunofluorescent staining for endothelial cell markers confirms endothelial cell identity. Non-senescent (NS), replicative senescent untreated, and replicative senescent treated cells stained for DAPI (blue; cell nuclei), CD146/vWF/CD144 (green; endothelial cell marker) and Phalloidin (red; F-Actin for cellular size) to confirm endothelial cell identity.
(PDF)

S2 Fig The treatment with VPA, Li_2_CO_3_, and tranilast does not influence cell function or morphology of non-senescent endothelial cells and hence does not regulate expression of OSKM. The treatment scheme was the same as for the replicative senescent cells. (A) Immunofluorescent staining for DAPI (blue; cell nuclei), CD31 (green; endothelial cell marker) and Phalloidin (red; F-Actin for cellular size). (B) Quantification of proliferation determined by BrdU incorporation assays. n = 3 (C) Determination of proliferation by total cell count in live-cell imaging over 24 hours. Cell count is expressed as fold change to time point 0h. n = 3 (D) Determination of migration capacity by a scratch-wound assay. n = 3 (E) Determination of angiogenic capacity by a tube formation assay and quantification of total branches length. n = 3 (F) Quantification of mRNA expression levels of OSKM by qRT-PCR 72-hours after treatment. n = 3 (G) qRT-PCR analysis of OSKM was performed 7 days after the 72-hour treatment period. n = 3.
(PDF)

S3 Fig The treatment with VPA, Li_2_CO_3_, and tranilast reduces DNA double strand breaks as indicated by γH2Ax but does not restore HMGB-1 levels. (A) Immunofluorescent staining of non-senescent (NS), replicative senescent untreated (RS untr) and replicative senescent treated (RS treated) cells for DAPI (blue) and γH2Ax (green) to quantify DNA double strand breaks. Green γH2Ax were quantified in relation to total cell count by DAPI. (B) Immunofluorescent staining of non-senescent (NS), replicative senescent untreated (RS untr) and replicative senescent treated (RS treated) cells for DAPI (blue) and HMGB-1 (green). Number of HMGB-1 positive cells was counted in relation to total cell count by DAPI.
(PDF)

S4 Fig The treatment with VPA, Li_2_CO_3_, and tranilast regulates ANG-1 and VEGF as pro-angiogenic markers. Quantification of angiogenic markers by qRT-PCR analysis. n = 3 *p < 0.05, ***p < 0.001.
(PDF)

S5 Fig Determination of migration capacity (A–D) and proliferation (E–H) after respective siRNA transfection with single siRNA for OSKM combined with the 72-hours treatment with VPA, Li_2_CO_3_, and tranilast. Migration was assessed by a scratch-wound assay and proliferation by live-cell count. Knockdown of a single component of OSKM by siRNA hindered the pro-migratory and pro-proliferative effect of the treatment with VPA, Li_2_CO_3_, and tranilast. n = 3 ***p < 0.001, ***p < 0.001, ****p < 0.0001.
(PDF)

S1 Table Used siRNAs for transfection. (PDF)

S2 Table Used primers for qRT-PCR. (PDF)

S3 Table Used antibodies. (PDF)

## Abbreviations

EC: Endothelial cells
HUVEC: Human umbilical vein endothelial cells
RS: Replicative senescent
iPSC: Induced pluripotent stem cell
Li_2_CO_3_: Lithium carbonate
NS: Non-senescent
OSKM: OCT3/4, SOX2, KLF4 and c-MYC
ROS: Reactive oxygen species
VPA: Valproic acid.
