# Supplementary material for: Restoration of angiogenic capacity in senescent endothelial cells by a pharmacological reprogramming approach
Source: PLoS One. 2025 Feb 28;20(2):e0319381. doi: 10.1371/journal.pone.0319381 (PMC11870368; doi:10.1371/journal.pone.0319381)

S1 Fig. Immunofluorescent staining for endothelial cell markers confirms endothelial cell identity. Non-senescent (NS), replicative senescent untreated, and replicative senescent treated cells stained for DAPI (blue; cell nuclei), CD146/vWF/CD144 (green; endothelial cell marker) and Phalloidin (red; F-Actin for cellular size) to confirm endothelial cell identity.

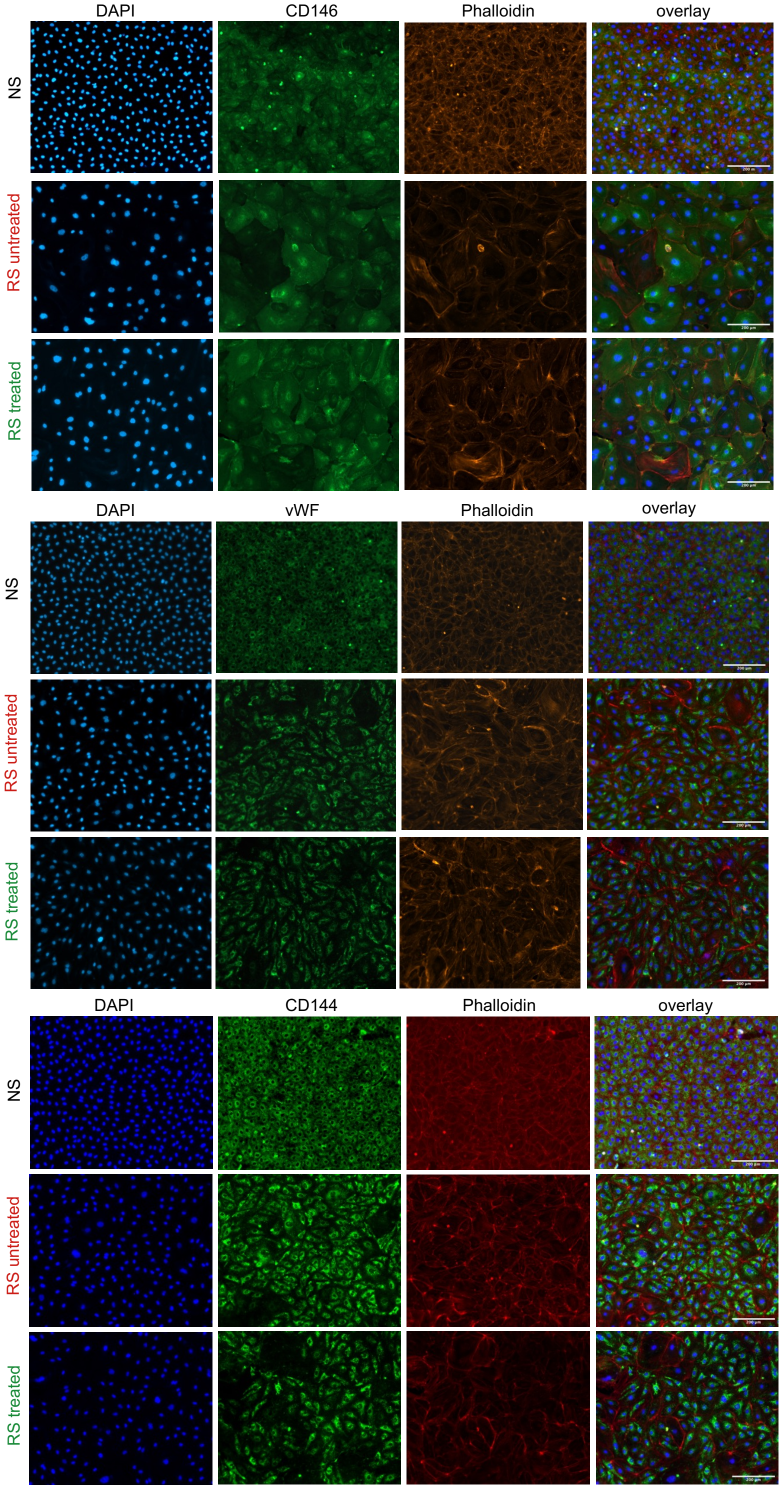

Supplement: S1 Fig — Non-senescent (NS), replicative senescent untreated, and replicative senescent treated cells stained for DAPI (blue; cell nuclei), CD146/vWF/CD144 (green; endothelial cell marker) and Phalloidin (red; F-Actin for cellular size) to confirm endothelial cell identity. (PDF) [file pone.0319381.s001.pdf]
