# Supplementary material for: Restoration of angiogenic capacity in senescent endothelial cells by a pharmacological reprogramming approach
Source: PLoS One. 2025 Feb 28;20(2):e0319381. doi: 10.1371/journal.pone.0319381 (PMC11870368; doi:10.1371/journal.pone.0319381)

S2 Fig. The treatment with VPA, Li2CO3, and tranilast does not influence cell function or morphology of non-senescent endothelial cells and hence does not regulate expression of OSKM. The treatment scheme was the same as for the replicative senescent cells. (A) Immunofluorescent staining for DAPI (blue; cell nuclei), CD31 (green; endothelial cell marker) and Phalloidin (red; F-Actin for cellular size). (B) Quantification of proliferation determined by BrdU incorporation assays. n=3 (C) Determination of proliferation by total cell count in live-cell imaging over 24 hours. Cell count is expressed as fold change to time point 0h. n=3 (D) Determination of migration capacity by a scratch-wound assay. n=3 (E) Determination of angiogenic capacity by a tube formation assay and quantification of total branches length. n=3 (F) Quantification of mRNA expression levels of OSKM by qRT-PCR 72-hours after treatment. n=3 (G) qRT-PCR analysis of OSKM was performed 7 days after the 72-hour treatment period. n=3

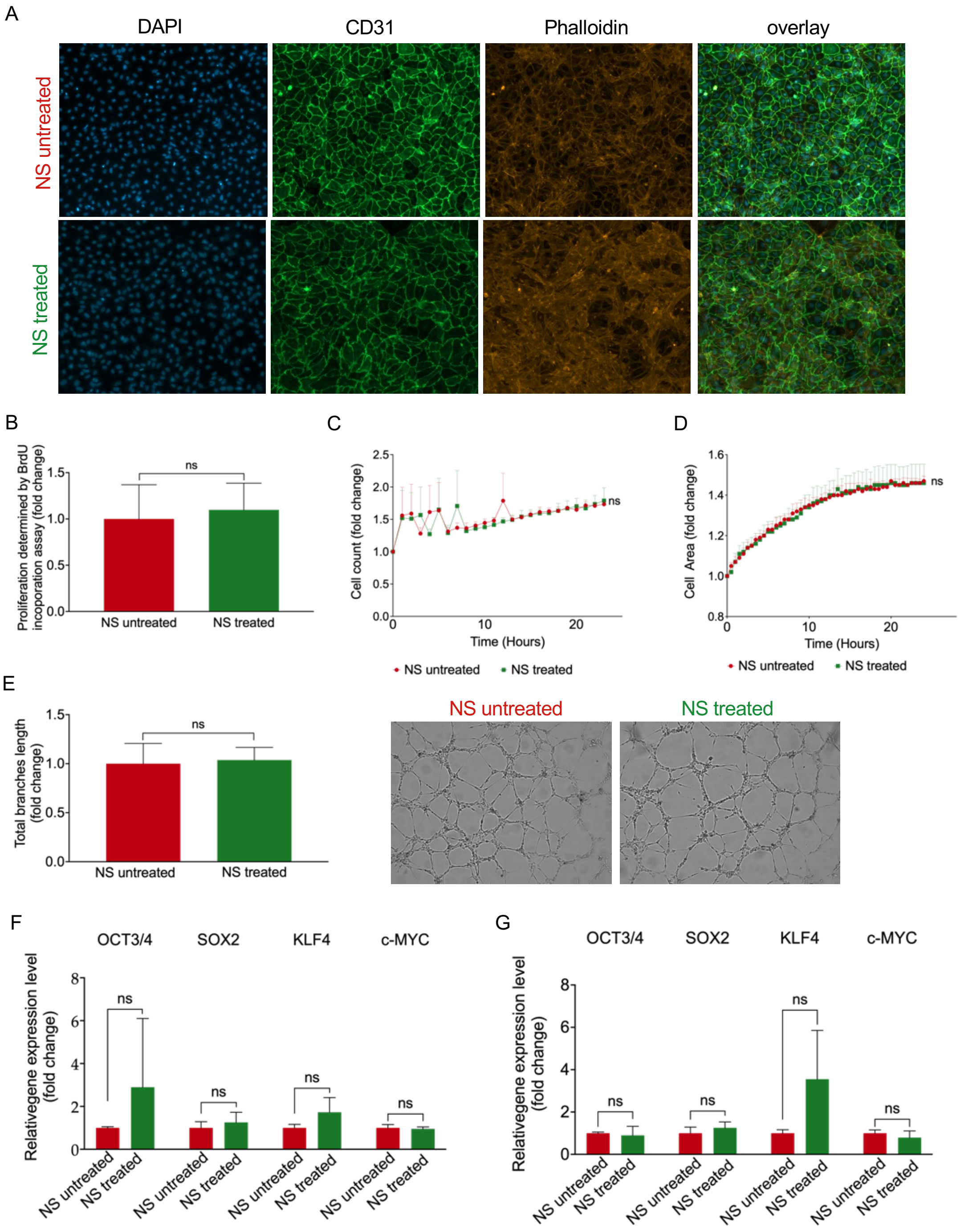

Supplement: S2 Fig — The treatment scheme was the same as for the replicative senescent cells. (A) Immunofluorescent staining for DAPI (blue; cell nuclei), CD31 (green; endothelial cell marker) and Phalloidin (red; F-Actin for cellular size). (B) Quantification of proliferation determined by BrdU incorporation assays. n = 3 (C) Determination of proliferation by total cell count in live-cell imaging over 24 hours. Cell count is expressed as fold change to time point 0h. n = 3 (D) Determination of migration capacity by a scratch-wound assay. n = 3 (E) Determination of angiogenic capacity by a tube formation assay and quantification of total branches length. n = 3 (F) Quantification of mRNA expression levels of OSKM by qRT-PCR 72-hours after treatment. n = 3 (G) qRT-PCR analysis of OSKM was performed 7 days after the 72-hour treatment period. n = 3. (PDF) [file pone.0319381.s002.pdf]
