# Supplementary material for: Restoration of angiogenic capacity in senescent endothelial cells by a pharmacological reprogramming approach
Source: PLoS One. 2025 Feb 28;20(2):e0319381. doi: 10.1371/journal.pone.0319381 (PMC11870368; doi:10.1371/journal.pone.0319381)

S3 Fig. The treatment with VPA, Li2CO3, and tranilast reduces DNA double strand breaks as indicated by γH2Ax but does not restore HMGB-1 levels. (A) Immunofluorescent staining of non-senescent (NS), replicative senescent untreated (RS untr) and replicative senescent treated (RS treated) cells for DAPI (blue) and γH2Ax (green) to quantify DNA double strand breaks. Green γH2Ax were quantified in relation to total cell count by DAPI. (B) Immunofluorescent staining of non-senescent (NS), replicative senescent untreated (RS untr) and replicative senescent treated (RS treated) cells for DAPI (blue) and HMGB-1 (green). Number of HMGB-1 positive cells was counted in relation to total cell count by DAPI.

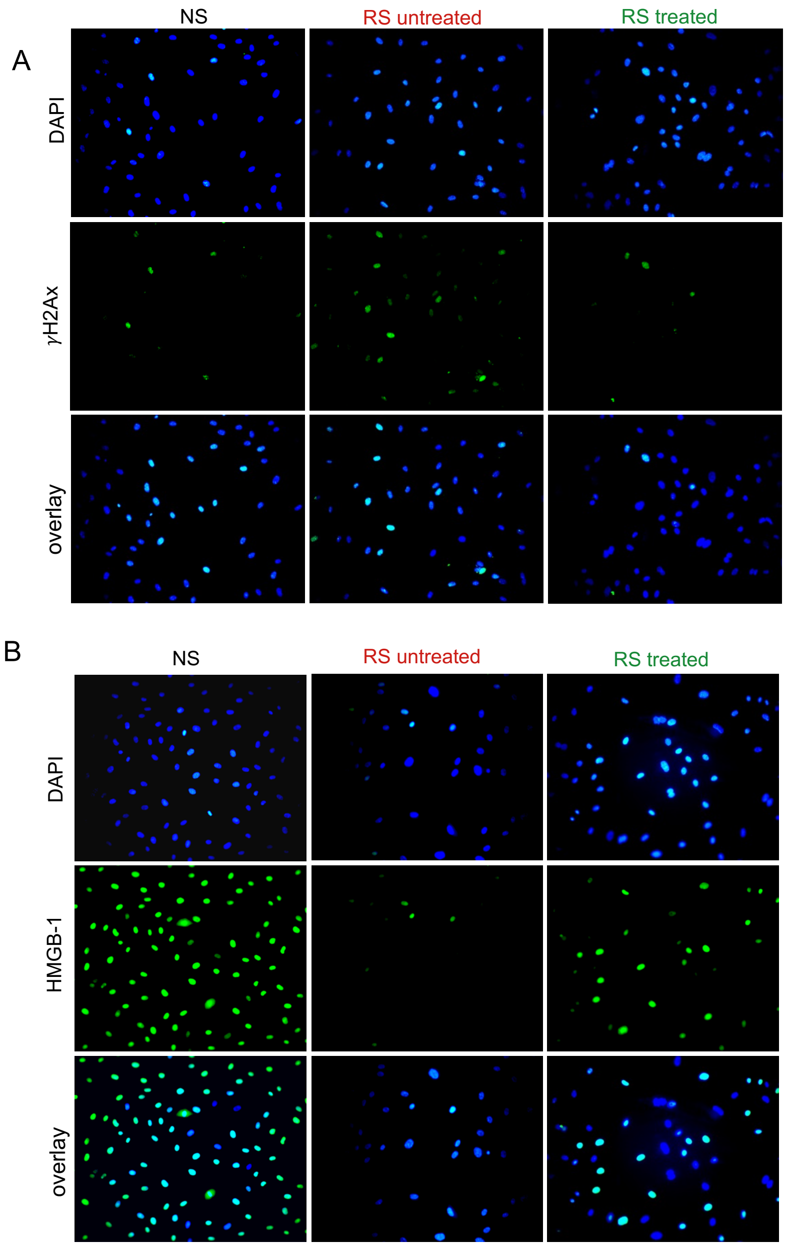

Supplement: S3 Fig — (A) Immunofluorescent staining of non-senescent (NS), replicative senescent untreated (RS untr) and replicative senescent treated (RS treated) cells for DAPI (blue) and γH2Ax (green) to quantify DNA double strand breaks. Green γH2Ax were quantified in relation to total cell count by DAPI. (B) Immunofluorescent staining of non-senescent (NS), replicative senescent untreated (RS untr) and replicative senescent treated (RS treated) cells for DAPI (blue) and HMGB-1 (green). Number of HMGB-1 positive cells was counted in relation to total cell count by DAPI. (PDF) [file pone.0319381.s003.pdf]
