# Supplementary material for: Restoration of angiogenic capacity in senescent endothelial cells by a pharmacological reprogramming approach
Source: PLoS One. 2025 Feb 28;20(2):e0319381. doi: 10.1371/journal.pone.0319381 (PMC11870368; doi:10.1371/journal.pone.0319381)

S4 Fig. The treatment regulates with VPA, Li2CO3, and tranilast regulates ANG-1 and VEGF as pro-angiogenic markers. Quantification of angiogenic markers by qRT-PCR analysis. n=3 *p<0.05, ***p<0.001.

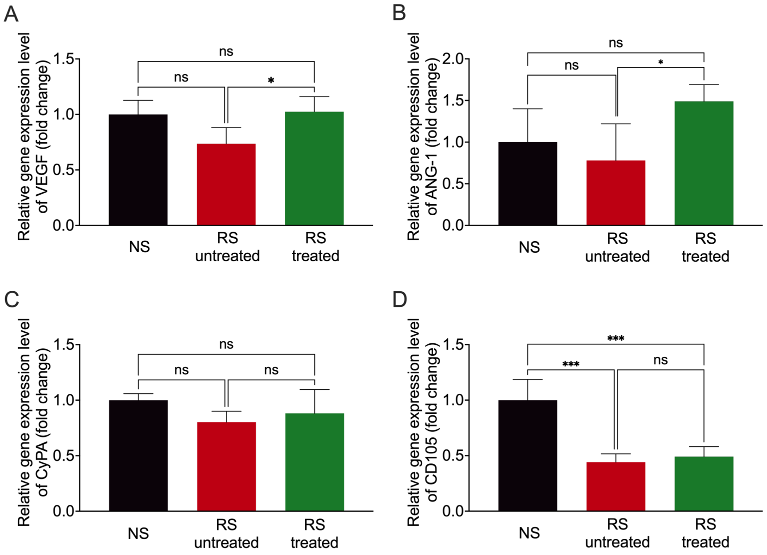

Supplement: S4 Fig — Quantification of angiogenic markers by qRT-PCR analysis. n = 3 *p < 0.05, ***p < 0.001. (PDF) [file pone.0319381.s004.pdf]
