# Supplementary material for: Restoration of angiogenic capacity in senescent endothelial cells by a pharmacological reprogramming approach
Source: PLoS One. 2025 Feb 28;20(2):e0319381. doi: 10.1371/journal.pone.0319381 (PMC11870368; doi:10.1371/journal.pone.0319381)

S5 Fig. Determination of migration capacity (A-D) and proliferation (E-H) after respective siRNA transfection with single siRNA for OSKM combined with the 72-hours treatment with VPA, Li2CO3, and tranilast. Migration was assessed by a scratch-wound assay and proliferation by live-cell count. Knockdown of a single component of OSKM by siRNA hindered the pro-migratory and pro-proliferative effect of the treatment with VPA, Li2CO3, and tranilast. n=3 *** p<0.001, *** p<0.001, **** p<0.0001.

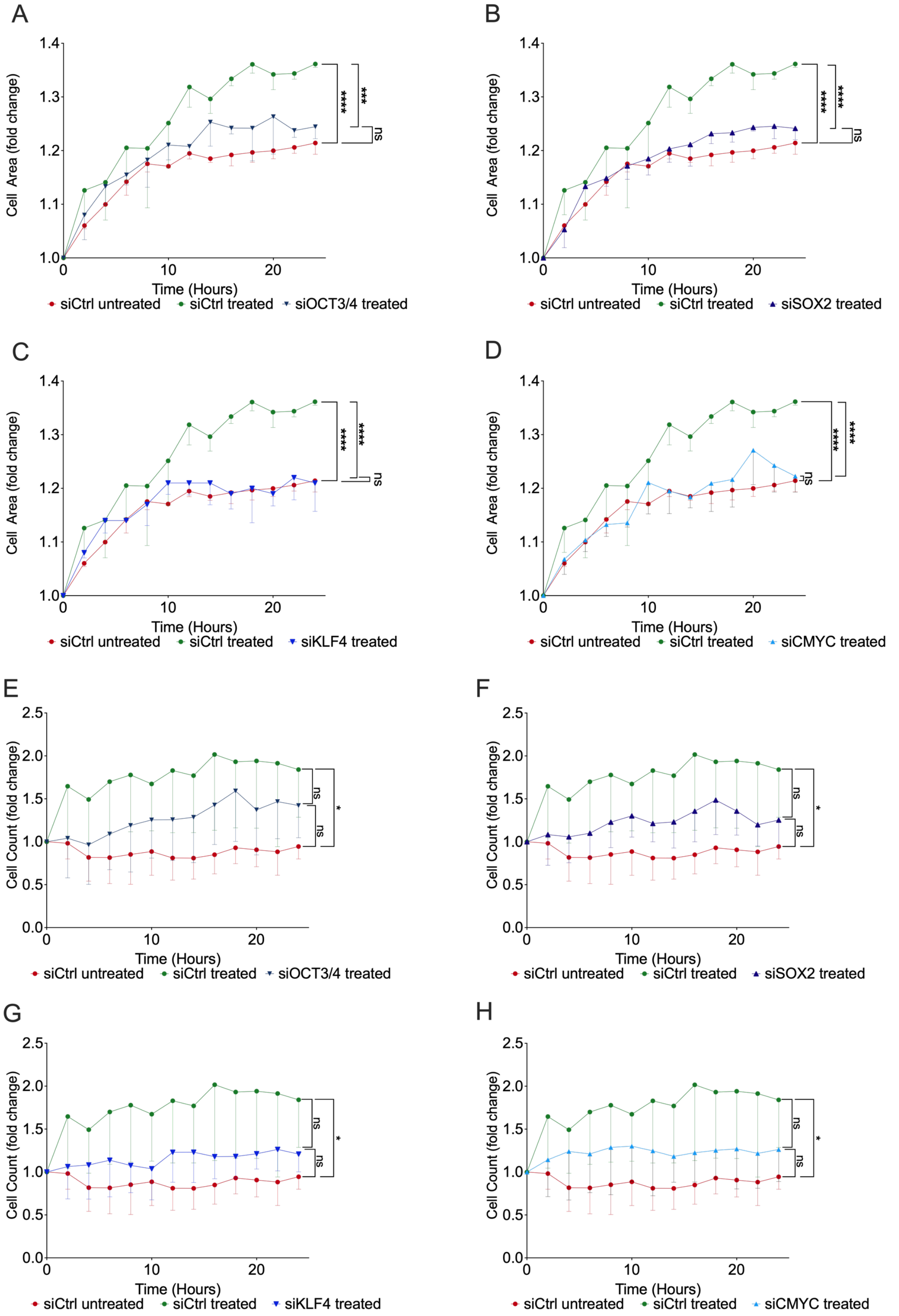

Supplement: S5 Fig — Migration was assessed by a scratch-wound assay and proliferation by live-cell count. Knockdown of a single component of OSKM by siRNA hindered the pro-migratory and pro-proliferative effect of the treatment with VPA, Li2CO3, and tranilast. n = 3 ***p < 0.001, ***p < 0.001, ****p < 0.0001. (PDF) [file pone.0319381.s005.pdf]
