# Supplementary material for: Restoration of angiogenic capacity in senescent endothelial cells by a pharmacological reprogramming approach
Source: PLoS One. 2025 Feb 28;20(2):e0319381. doi: 10.1371/journal.pone.0319381 (PMC11870368; doi:10.1371/journal.pone.0319381)
Supplement: S1 Table — (PDF) [file pone.0319381.s006.pdf]

S1 Table: Used siRNAs for lipotransfection

Target gene	siRNA name	Order number	Manufacturer
c-MYC	ON-TARGET plus Human MAC (4609) siRNA – SMARTpool	L-003282-02-0005	Dharmacon, Horizon Discovery, Lafayette, US
KLF4	ON-TARGET plus Human KLF4 (9314) siRNA - SMARTpool	L-005089-00-0005	Dharmacon, Horizon Discovery, Lafayette, US
POU5F1 (OCT3/4)	Hs_POU5F1_2 Flexitube siRNA	SI00690382	Qiagen, Hilden, Germany
SOX2	Hs_SOX2_5 Flexitube siRNA	SI04136167	Qiagen, Hilden, Germany
Scrambled control	ON-TARGET plus Non-targeting Pool	D-001810-10-05	Dharmacon, Horizon Discovery, Lafayette, US
