# Supplementary material for: Restoration of angiogenic capacity in senescent endothelial cells by a pharmacological reprogramming approach
Source: PLoS One. 2025 Feb 28;20(2):e0319381. doi: 10.1371/journal.pone.0319381 (PMC11870368; doi:10.1371/journal.pone.0319381)
Supplement: S2 Table — (PDF) [file pone.0319381.s007.pdf]

S2 Table: Used primers for qRT-PCR.

Gene		Sequence
OCT3/4	for	AGC GAA CCA GTA TCG AGA AC
	rev	TTA CAG AAC CAC ACT CGG AC
SOX2	for	GGG AAA TGG GAG GGG TGC AAA AGA GG
	rev	TTG CGT GAG TGT GGA TGG GAT TGG TG
CD44	for	TGGCACCCGCTATGTCGAG
	rev	GTAGCAGGGATTCTGTCTG
TNFα	for	GGCGTGGAGCTGAGAGATA
	rev	CAGCCTTGGCCCTTGAAGA
IL1β	for	AAAGCTTGGTGATGTCTGGTC
	rev	GGACATGGAGAACACCACTTG
IL-6	for	GGC ACT GGC AGA AAA CAACC
	rev	GCA AGT CTC CTC ATT GAA TCC
IL-8	for	GAGAGTGATTGAGAGTGGACCAC
	rev	CACAACCCTCTGCACCCAGTTT
p16INK4A	for	GGG GGC ACC AGA GGC AGT
	rev	GGT TGT GGC GGG GGC AGT T
p53	for	CCTCAGCATCTTATCCGAGTGG
	rev	TGGATGGTGGTACAGTCAGAGC
p15	for	ACGGAGTCAACCGTTTCGGGAG
	rev	GGTCGGGTGAGAGTGGCAGG
p21	for	TGT CCG TCA GAA CCC ATG C
	rev	AAA GTC GAA GTT CCA TCG CTC
KLF4	for	TCT CAA GGC ACA CCT GCG AA
	rev	TAG TGC CTG GTC AGT TCA TC
GAPDH	for	TGCACCACCAACTGCTTAGC
	rev	GGCATGGACTGTGGTCATGAG

FBX07	for	GCTCGCACCTGAGGCAGTCC
	rev	GTCTCTTCATCTCCAGTGAGGGG
c-MYC	for	GCG TCC TGG GAA GGG AGA TCC GGA GC
	rev	TTG AGG GGC ATC GTC GCG GGA GGC TG
CyPA	for	CCCACCGTGTTCTTCGACATT
	rev	GGACCCGTATGCTTTAGGATGA
ANG-1	for	AGCGCCGAAGTCCAGAAAAC
	rev	TACTCTCACGACAGTTGCCAT
VEGF	for	CTACCTCCACCATGCCAAGT
	rev	GCAGTAGCTGCGCTGATAGA
CD105	for	TGCACTTGGCCTACAATTCCA
	rev	AGCTGCCCACTCAAGGATCT
