# Supplementary material for: Restoration of angiogenic capacity in senescent endothelial cells by a pharmacological reprogramming approach
Source: PLoS One. 2025 Feb 28;20(2):e0319381. doi: 10.1371/journal.pone.0319381 (PMC11870368; doi:10.1371/journal.pone.0319381)
Supplement: S3 Table — (PDF) [file pone.0319381.s008.pdf]

S3 Table: Used antibodies

Target	Order number	Dilution	Manufacturer
CD31	#3528S	1:1000	Cell signalling
CD146	ab75769	1:500	abcam
vWF	ab6994	1:500	abcam
VE-cadherin	555289	1:500	BD Pharmingen
Phalloidin-TRITC	P1951	1:100	Sigma Aldrich
